# Spread of Multidrug-Resistant Enterobacterales Strains Isolated From the Hospital Environment and Clinical Specimens Within ICUs in Tunisia

**DOI:** 10.7759/cureus.102231

**Published:** 2026-01-24

**Authors:** Sana Bahri, Meriem Souguir, Sana Azaiez, Asma Ben Cheikh, Hela Ghali, Raoudha Chrigui, Mohamed Sahbi Chelbi, Lamia Tilouche, Meriem Mtibâa, Tounes Ben Romdhane, Houyem Said, Olfa Bouallegue, Wejdene Mansour

**Affiliations:** 1 Laboratory of Metabolic Biophysics and Applied Pharmacology, Ibn Al Jazzar Faculty of Medicine, University of Sousse, Sousse, TUN; 2 Faculty of Medicine, University of Sousse, Sousse, TUN; 3 Department of Prevention and Security of Care, University Hospital of Sousse, Sousse, TUN; 4 Surgical Intensive Care Unit, University Hospital of Sousse, Sousse, TUN; 5 Sahloul Microbiology Laboratory, University Hospital of Sousse, Sousse, TUN; 6 Department of Pharmacy, Sahloul University Hospital, Sousse, TUN; 7 Department of Prevention and Security of Care, Sahloul University Hospital, Sousse, TUN; 8 Research Laboratory: Emerging Bacterial Resistance in Hospital, Veterinary and Environmental Settings: Impact on the Safety of Care (LR20SP06), Faculty of Medicine, University of Sousse, Sousse, TUN

**Keywords:** ctx-m-15, enterobacterales, hospital environment, intensive care units, ndm-1, oxa-204

## Abstract

The hospital environment is a recognized reservoir for multidrug-resistant (MDR) bacteria, and inadequate hygiene practices by healthcare workers (HCWs) may contribute to the transmission and persistence of healthcare-associated infections (HAIs), particularly in ICUs. This study investigated the presence, genetic characteristics, and transmission pathways of MDR Enterobacterales in the environment and among HCWs in three ICUs, such as surgical (ICU-S), medical (ICU-M), and pediatric (ICU-P), at Sahloul Hospital, Sousse, Tunisia. From September to December 2020, 140 samples were collected from environmental surfaces (n = 114) and HCWs’ hands (n = 26). Of these, nine samples (6.42%) tested positive for resistant Enterobacterales, yielding 15 resistant isolates, including one *Klebsiella pneumoniae* and eight *Enterobacter cloacae*. Notably, no resistant Enterobacterales were recovered from HCWs’ hand samples. Whole-genome sequencing was performed on seven isolates (five clinical and two environmental) to assess genetic relatedness and resistance profiles. Three distinct sequence types (STs) of *K. pneumoniae* were identified: ST147 (clinical and environmental), ST307, and ST353. The ST147 isolate from an antiseptic bottle in ICU-M co-produced CTX-M-15 and NDM-1 enzymes and carried the biocide resistance gene *qacE*. Despite both being ST147, the environmental and clinical isolates were not clonal (140 single-nucleotide polymorphism (SNP) differences). The ST307 isolate from a blood sample co-produced CTX-M-15 and OXA-204. Among *E. cloacae* isolates, ST344 (n = 2) was identified from two different ICUs and differed by only 6 SNPs, suggesting inter-ICU transmission. These isolates harbored *bla*ACT-16, *bla*CTX-M-15, and multiple other resistance genes. One environmental *E. cloacae* isolate belonged to the clone ST78 and was recovered from a soap dispenser in ICU-P, carrying *blaACT-15* and *fosA*. These findings underscore the potential of contaminated surfaces to harbor and disseminate MDR Enterobacterales. Strengthened infection control measures, environmental monitoring, and genomic surveillance are essential to reduce HAIs in ICU settings.

## Introduction

Healthcare-associated infections (HAIs) caused by Enterobacterales are a significant concern in healthcare settings worldwide. In North Africa, including Tunisia, multidrug-resistant (MDR) Enterobacterales, particularly carbapenemase-producing *Klebsiella pneumoniae *and *Enterobacter cloacae*, have been increasingly reported in both clinical and hospital environmental settings. This trend poses significant challenges to infection prevention and control and justifies targeted local investigations.

Enterobacterales are a family of bacteria that commonly inhabit the gastrointestinal tract of humans and animals. While many strains are harmless, some species within this family, such as *Escherichia coli*, *K. pneumoniae*, and *Enterobacter *species, can cause infections, particularly in individuals with weakened immune systems or those undergoing medical procedures. Among the leading global threats to public health, carbapenemase-producing Enterobacterales, especially *K. pneumoniae *and *E. cloacae*, are considered some of the top international threats [1].

Transmission of Enterobacterales in healthcare settings can occur through various routes, including contact with contaminated surfaces, medical equipment, or healthcare personnel or the ingestion of contaminated food or water. Factors contributing to the prevalence of HAIs caused by Enterobacterales include the overuse and misuse of antibiotics, inadequate infection control measures, and the presence of MDR strains.

Although direct patient infection resulting from contact with inanimate surfaces may be relatively uncommon, contaminated surfaces represent critical environmental reservoirs for Enterobacterales in healthcare settings. These reservoirs facilitate high-risk indirect transmission through contact with medical equipment, instruments, and, importantly, the hands of healthcare workers (HCWs), thereby contributing to the amplification and persistence of HAIs. Effective environmental hygiene and appropriate handling practices are therefore essential to interrupt transmission pathways, particularly in ICUs.

Despite the widespread use of disinfectants in healthcare settings, the emergence of biocide resistance determinants and/or improper disinfection practices may compromise their effectiveness and contribute to the persistence of MDR Enterobacterales in the hospital environment [2].

The aim of this study was to explore how medical equipment, furniture, hospital personnel, and patients contribute to the spread of resistant Enterobacterales clones within a Tunisian hospital over a four-month period. We employed next-generation sequencing technologies to examine the genetic relatedness of selected isolates at the genome level. Additionally, we characterized the presence of resistance genes and associated genetic elements.

The primary objective of this study was to investigate the patterns of environmental and clinical dissemination of MDR Enterobacterales within three ICUs at Sahloul Hospital. Secondary objectives included molecular characterization of the isolates, analysis of their antimicrobial resistance profiles, and assessment of clonal relatedness using pulsed-field gel electrophoresis (PFGE) and whole-genome sequencing (WGS). Our findings provide a comprehensive molecular analysis of Enterobacterales isolates and emphasize the importance of implementing strict hygienic protocols to control pathogen dissemination in healthcare settings.

## Materials and methods

Sample collection, isolation, and identification of resistant Enterobacterales from ICU settings

From September to December 2020, a cross-sectional study was conducted in three of the five ICUs at Sahloul University Hospital in Sousse, Tunisia. In the ICUs (n = 3), 35 nurses and medical staff attended to 17 patient beds. Bed distribution was as follows: ten for the general surgery ICU (ICU-S), four for the medical ICU (ICU-M), and three for the pediatric ICU (ICU-P). Sliding curtains separated patient beds in each ICU. Samples were collected by swabbing items and medical equipment using sterile swabs (eSwab, Copan, Murrieta, USA). These items were located in three ICU wards across two floors of the hospital: ICU-S (n = 69, comprising 56 environmental and 13 personnel samples), ICU-P (n = 34, comprising 26 environmental and eight personnel samples), and ICU-M (n = 37, comprising 32 environmental and five personnel samples). Environmental samples (n = 114) targeted surfaces frequently touched by both patients and healthcare professionals, including door handles, patient beds, stethoscopes, liquid soap dispensers, and medical devices. Hand swabs were collected from HCWs (n = 26).

Swabs collected from the hospital environment and staff hands were incubated for 24 hours in nutrient broth for enrichment. Following enrichment, a drop of culture was streaked onto MacConkey agar plates supplemented with cefotaxime or imipenem (2 mg/L), and plates were incubated overnight at 37 °C. One colony per morphology and per plate was selected and identified using API 20E galleries (bioMérieux, Marcy-l’Étoile, France). All identified isolates were stored at −80 °C for further analyses.

Seven clinical strains of Enterobacterales (four *K. pneumoniae *and three *E. cloacae*) were retrieved from the strain library of the hospital bacteriology laboratory. These strains were originally isolated from patients hospitalized at CHU Sahloul during the same period. Specifically, two *E. cloacae *strains were isolated from ICU-S, while ICU-M contributed two *K. pneumoniae *and one *E. cloacae *strain, and ICU-P contributed two *K. pneumoniae *strains. These clinical strains were used for comparative analyses (Table 1).

**Table 1:** Clinical features of patients with K. pneumoniae and E. cloacae strains isolated in ICUs (September to December 2020) ICU-M, ICU-medical; ICU-P, ICU-pediatric; ICU-S, ICU-surgery

Identifier	Strain	Year of birth	Ward	Type of sample	Date of hospitalization	Date of sampling	Reason for hospitalization	Outcome
58665	Klebsiella pneumoniae	2019	ICU-P	Blood	08/05/2020	09/16/2020	Congenital heart disease (tetralogy of Fallot)	Left
58666	K. pneumoniae	-	ICU-P	Blood	09/23/2020	-	-	-
58530	K. pneumoniae	-	ICU-M	Blood	-	11/14/2020	-	-
58540	Enterobacter cloacae	1972	ICU-S	Distal sampling	11/13/2020	11/16/2020	Polytrauma	Died
58552	E. cloacae	1972	ICU-S	Blood	09/23/2020	10/08/2020	Brain tumor	Died
58531	E. cloacae	-	ICU-M	Catheter	-	11/24/2020	-	-
58532	K. pneumoniae	-	ICU-M	Tracheal	-	12/25/2020	-	-

Antimicrobial susceptibility testing

Antimicrobial susceptibility testing was performed using the disk diffusion method on Mueller-Hinton agar plates, following EUCAST 2021 guidelines. A total of 16 β-lactams (including penicillins, β-lactam/β-lactamase inhibitor combinations, first- to fourth-generation cephalosporins, aztreonam, and ertapenem) and 15 non-β-lactams (aminoglycosides, tetracyclines, quinolones, and other classes) were tested (full list and concentrations provided in Appendix A). *E. coli *ATCC 25922 served as the control strain. Phenotypic detection of extended-spectrum β-lactamase (ESBL) production was carried out using the double-disc synergy test.

Strains clonality by PFGE

All *K. pneumoniae *and *E. cloacae *isolates underwent PFGE using the XbaI restriction enzyme to determine their genetic relatedness. Electrophoresis was performed on a CHEF Mapper XP system at 6 V/cm and 14 °C for 24 hours, with pulse times ranging from 10 to 60 seconds and an angle of 120°. PFGE patterns were interpreted according to the criteria of Tenover et al. [3]. Restriction fragments were analyzed using GelCompar II software 6.5 (Applied Maths, Austin, TX, USA), and dendrograms were constructed using the unweighted pair group method with arithmetic mean based on the Dice similarity index.

PFGE was initially performed to assess clonality among the collected isolates. Based on PFGE patterns, seven representative strains were selected for WGS, ensuring that genetically distinct isolates were included. Subsequent WGS data were analyzed in silico using tools such as ResFinder to identify antimicrobial resistance genes.

Detection of resistance genes by PCR

Bacterial DNA was extracted from freshly cultivated pure cultures on standard growth media using thermal lysis. PCR assays were performed to detect the following genes: *blaCTX-M *genes were identified using a CTX-M group-specific multiplex PCR [4], and the carbapenemase genes *blaOXA-48-like*, *blaNDM*, and *blaKPC *were detected using a multiplex PCR.

PCR products were analyzed using the QIAxcel Advanced automatic DNA/RNA analyzer (Qiagen, Hilden, Germany), following the manufacturer’s instructions. All 15 initial MDR Enterobacterales isolates were first screened by wet-lab PCR to identify key antimicrobial resistance genes prior to WGS selection. Based on PFGE patterns and epidemiological relevance, seven isolates, including both environmental and clinical strains, were selected for WGS. Subsequent WGS data were analyzed in silico using tools such as ResFinder to confirm and expand the PCR-based resistance gene profiles.

WGS and bioinformatics analysis

Based on the PFGE results, five clinical and two environmental isolates were selected for short-read sequencing. DNA was extracted using the NucleoSpin Microbial DNA extraction kit (Macherey-Nagel, Hoerdt, France). Libraries were prepared using Nextera XT technology, and sequencing was performed on a NovaSeq 6000 instrument (Illumina, San Diego, CA, USA).

Quality control of the reads was conducted using FastQC, and low-quality sequences were trimmed using Trimmomatic v0.39. Reads were then assembled de novo using Shovill 1.0.0, and the quality of the assemblies was assessed using QUAST v4.5. Sequence types (STs), resistance genes, and virulence factors were determined using CGE online tools, including MLSTFinder v2.0, ResFinder v4.1, and VirulenceFinder 2.0.3 (www.genomicepidemiology.org). All raw sequencing reads and assembled genomes generated in this study have been deposited in the European Nucleotide Archive under BioProject accession number PRJNA1400037, ensuring full transparency and reproducibility of the genomic analyses.

Phylogenetic analysis

The pan-genome was determined, and a core genome of the WGS collection was generated using Roary v3.13.0, with a protein BLAST identity threshold of 80% and a core definition of 90%. Prokka annotations were provided to Roary as input. Recombination was subsequently removed using Gubbins v3.2.1, and a maximum likelihood tree was constructed from the core-gene alignment using RAxML v7.7.6 with default parameters. Pairwise single-nucleotide polymorphism (SNP) distances were calculated from the core-genome alignments generated by Roary using snp-dists v0.8.2 on the Galaxy Europe platform. Results are provided in the Appendices. The resulting phylogenetic tree of *K. pneumoniae *isolates was visualized using iTOL v7 (itol.embl.de/itol.cgi).

Ethical considerations

The study protocol was approved by the Ethics Committee and the hospital administration of Sahloul Hospital. All procedures involving environmental sampling, HCWs’ hand sampling, and the use of clinical isolates were conducted in accordance with institutional regulations and ethical guidelines (approval HS 27-2023).

## Results

Epidemiological data

In 2020, 450 strains of *K. pneumoniae *and 175 strains of *E. cloacae *were isolated in the Microbiology Department at Sahloul Hospital, Sousse. These strains originated from all wards of the hospital. Eleven, 41, and 10 strains were isolated from the ICUs ICU-M, ICU-S, and ICU-P, respectively (Table 2).

**Table 2:** Number of K. pneumoniae and E. cloacae strains isolated in ICUs at Sahloul University Hospital, Sousse, Tunisia (2020) CTX, cefotaxime; ETP, ertapenem; ICU-M, ICU-medical; ICU-P, ICU-pediatric; ICU-S, ICU-surgery; R, resistant

Ward	Number of* Klebsiella pneumoniae*	CTX^R^*K. pneumoniae *(sequenced strains)	ETP^R^* K. pneumoniae*	Number of *Enterobacter cloacae*	CTX^R^ *E. cloacae *(sequenced strains)	ETP^R^ *E. cloacae*	Total
ICU-M	10	9 (1)	6 (1)	1	1 (1)	0	11
ICU-S	22	11	4	19	3 (1)	1	41
ICU-P	8	8 (1)	7	2	0	0	10
Total	40	28	17	22	3	1	62

The overall percentage of resistance to cefotaxime across the three ICU wards (ICU-S, ICU-M, ICU-P) was 50% (31/62), while the rate of resistance to ertapenem was 29% (18/62). Resistance rates in *K. pneumoniae *were substantially higher than in *E. cloacae*: 70% versus 13.6% for cefotaxime and 42.5% versus 4.5% for ertapenem, respectively. Among the ICUs, ICU-S exhibited the highest percentages of resistance to both antibiotics (Table 3).

**Table 3:** Characterization of strains isolated from the ICU environment and from patients included in the study Cells in bold correspond to strains for which the whole genome has been sequenced. AMC, amoxicillin + clavulanic acid; AMX, amoxicillin; AN, amikacin; APR, apramycin; ATM, aztreonam; CAZ, ceftazidime; CF, cefalotin; CFP, cefquinome; C, chloramphenicol; CTX, cefotaxime; CXM, cefuroxime; EFT, ceftiofur; ENR, enrofloxacin; ETP, ertapenem; FEP, cefepime; FFC, florfenicol; FOX, cefoxitin; GEN, gentamicin; ICU-M, ICU-medical; ICU-P, ICU-pediatric; ICU-S, ICU-surgery; K, kanamycin; NA, nalidixic acid; NET, netilmicin; OFX, ofloxacin; PIP, piperacillin; TE, tetracycline; TM, tobramycin; TIC, ticarcillin; TCC, ticarcillin + clavulanic acid; CS, colistin; S, streptomycin; SUL, sulfamethoxazole; TMP, trimethoprim; TZP, piperacillin + tazobactam

Group	Ref	Strain	Source	Ward (floor)	Sampling date	Antibiogram																																
						AMX	CFP	PIP	TIC	FEP	AMC	CAZ	TZP	CF	EFT	TCC	ATM	CXM	FOX	CTX	ETP	K	TM	GEN	APR	S	AN	NET	TE	C	FFC	CS	NA	ENR	OFX	TMP	SUL	Beta-lactamases
Environmental strains	58423	Klebsiella pneumoniae	Antiseptic bottle	ICU-M (ground)	10/15/2020	R	I	R	R	I	R	R	R	R	R	R	R	R	R	R	I	I	R	R	S	S	S	I	R	S	S	I	I	R	R	R	R	CTX-M1, NDM-1
	58470	Enterobacter cloacae	Electricity switch	ICU-S (first)	11/18/2020	I	S	I	I	S	R	I	S	R	I	I	S	R	R	I	S	S	S	S	S	S	S	S	S	S	S	I	S	S	S	S	S	-
	58471	E. cloacae	Respirator		11/18/2020	R	S	I	I	S	R	I	I	R	R	R	I	R	R	R	S	S	S	S	S	S	S	S	I	S	S	S	S	S	S	S	S	-
	58480	E. cloacae	Syringe plunger		11/18/2020	R	S	S	S	S	R	S	S	R	S	S	S	S	I	I	S	S	S	S	S	I	S	S	S	S	S	S	S	S	S	S	S	-
	58490	E. cloacae	Patient bed		11/18/2020	R	S	S	S	S	R	S	S	R	S	S	S	S	R	I	S	S	S	S	S	S	S	S	S	S	S	I	S	S	S	S	S	-
	58506	E. cloacae	Hand’s sample		11/18/2020	R	S	S	S	S	R	S	S	R	S	S	S	S	I	I	S	S	S	S	S	I	S	S	R	S	S	S	S	S	S	R	R	-
	58507	E. cloacae	Hand’s sample		11/18/2020	R	S	I	S	S	I	I	I	R	I	S	S	R	I	I	S	S	S	S	S	S	S	S	R	S	S	S	S	S	S	S	S	-
	58500	*Pantoae *sp.	Medicine cabinet		11/18/2020	I	S	I	S	S	I	S	S	R	I	S	I	I	R	I	S	S	S	S	S	I	S	S	S	R	R	S	S	S	S	I	S	-
	58513	E. cloacae	Soap dispenser	ICU-P (first)	12/02/2020	R	S	R	R	S	R	I	I	R	R	R	I	R	I	R	S	S	S	S	S	S	S	S	S	S	S	S	S	S	S	S	S	-
	58514	E. cloacae	Soap dispenser		12/02/2020	R	S	S	S	S	I	S	S	R	R	S	S	S	R	I	S	S	S	S	S	I	S	S	S	S	S	S	S	S	S	S	S	ACT-5
	58515	Providencia rettgeri	Doorknob		12/02/2020	R	S	I	S	S	R	S	S	R	S	S	S	I	S	I	S	I	I	I	S	I	S	S	R	R	R	-	I	I	I	R	R	CMY
	58516	P. rettgeri	Doorknob		12/02/2020	I	S	S	S	S	S	S	S	I	S	S	S	S	S	I	S	I	I	I	S	I	S	S	R	R	S	-	I	I	I	R	R	CMY
	58517	P. rettgeri	Drawer		12/02/2020	R	S	S	S	S	I	S	S	I	S	S	S	S	S	I	S	I	I	I	S	I	S	S	R	R	R	-	I	I	I	R	R	CMY
	58519	*Pantoae *sp.	Antiseptic bottle		12/02/2020	R	S	S	R	S	S	S	S	S	S	S	S	S	S	I	S	S	S	S	S	S	S	S	S	S	S	S	S	S	S	S	S	-
Clinical samples	58530	K. pneumoniae	Blood	ICU-M (ground)	11/14/2020	R	I	R	R	I	R	I	I	R	I	R	I	R	S	I	S	I	R	I	S	R	S	I	R	S	S	I	R	R	R	R	R	OXA-48-like, CTX-M1
	58531	E. cloacae	Urine Catheter		11/24/2020	I	I	R	R	S	R	I	S	R	I	I	I	R	R	I	S	I	I	I	S	I	S	I	R	R	S	I	I	S	S	R	R	CTX-M1
	58532	K. pneumoniae	Tracheal tube		12/25/2020	R	S	I	R	S	R	S	S	R	S	I	S	R	R	I	S	S	S	S	S	I	S	S	S	S	S	I	I	I	S	S	S	SHV, DHA
	58540	E. cloacae	Distal sampling	ICU-S (first)	11/16/2020	I	S	S	S	S	I	S	S	R	S	S	S	I	I	I	S	S	S	S	S	I	S	S	R	R	I	I	R	I	I	R	I	-
	58552	E. cloacae	Blood		10/08/2020	R	I	R	R	I	R	I	S	R	I	I	I	R	R	I	S	I	I	I	S	R	S	I	I	R	S	I	S	S	S	R	R	CTX-M1
	58665	K. pneumoniae	Blood	ICU-P (first)	09/16/2020	R	R	R	R	I	R	R	R	R	R	R	R	R	R	R	I	R	I	S	S	I	I	I	I	S	S	I	R	R	R	S	R	-
	58666	K. pneumoniae	Blood		09/23/2020	R	S	I	R	S	I	S	S	S	S	I	S	S	S	I	S	R	I	S	S	I	I	I	I	S	S	I	R	I	I	S	S	SHV

Sample collection and bacterial identification from the hospital environment

A total of 140 samples were collected from three ICU wards between September and December 2020. Nine samples tested positive, yielding 14 resistant strains: one *K. pneumoniae* was isolated from an antiseptic bottle in ICU-M. Two *E. cloacae *strains were recovered from two separate soap dispensers in ICU-P, while six *E. cloacae* strains were isolated in ICU-S from various sources: medical staff (n = 2), respirator (n = 1), syringe plunger (n = 1), patient bed (n = 1), and an electrical switch (n = 1) (Table 3).

Five additional Enterobacterales were identified: two *Pantoea* sp. strains (from a medicine cabinet and an antiseptic bottle) and three *Providencia rettgeri *strains, one from a drawer and two from a doorknob. Notably, two *E. cloacae *strains were recovered from the hands of healthcare personnel in ICU-S, highlighting a potential transmission route and underscoring the role of staff in the dissemination of MDR Enterobacterales within the ICUs. Results of the antimicrobial susceptibility testing are summarized in Table 3.

Molecular characterization of antimicrobial resistance genes by PCR

The ESBL CTX-M-1-like gene was detected in two *K. pneumoniae *strains (one clinical and one environmental) and in two clinical *E. cloacae *strains. One clinical *K. pneumoniae *strain expressed the extended-spectrum cephalosporinase *blaDHA*, while the three environmental *P. rettgeri *strains expressed *blaCMY*. The NDM-1 carbapenemase was detected in an environmental *K. pneumoniae *strain, and the OXA-48-like carbapenemase was detected in one *K. pneumoniae *strain, although this isolate remained susceptible to ertapenem.

Strains typing by PFGE

**Figure 1:**
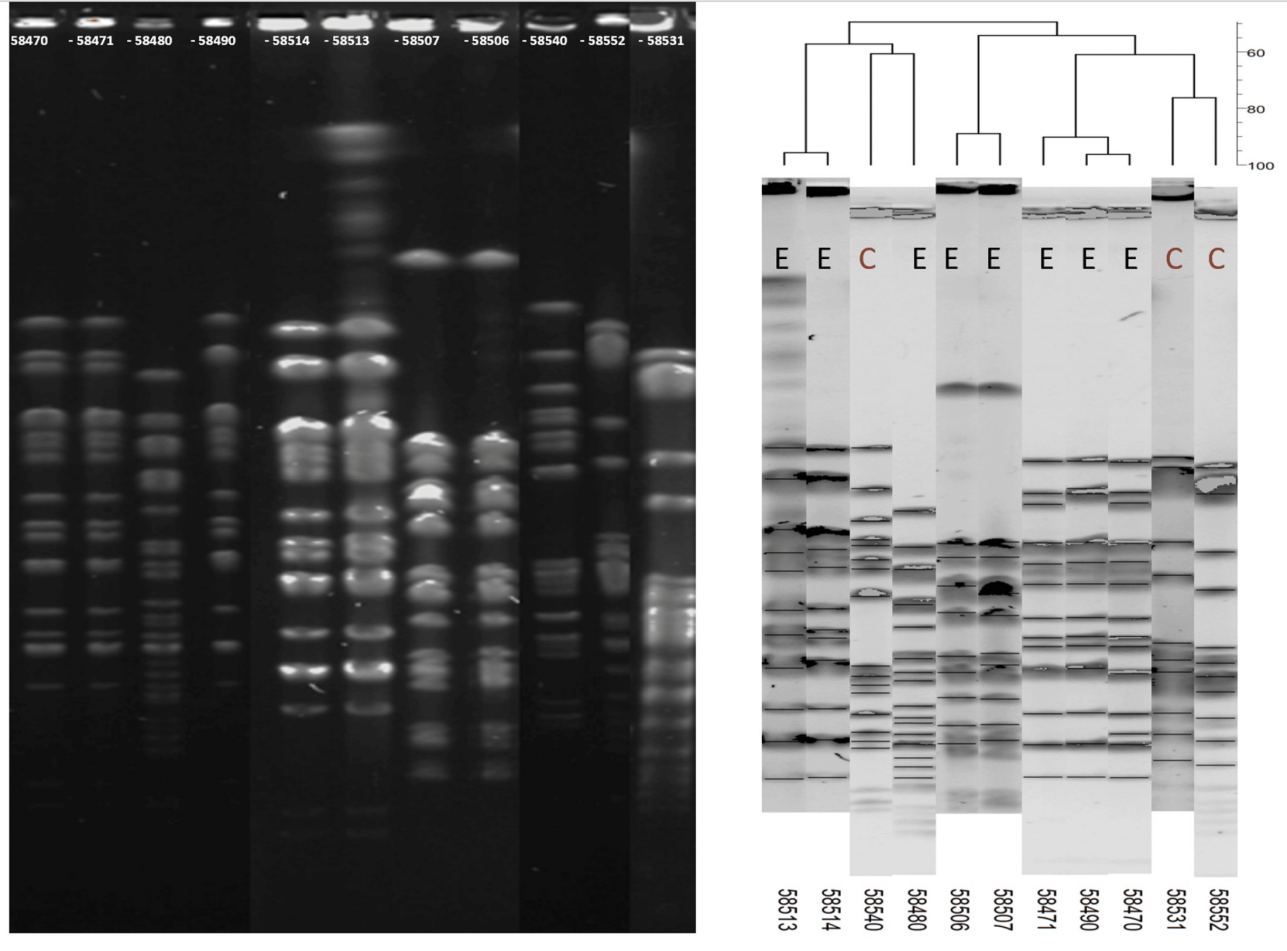
PFGE-based phylogeny of Enterobacter cloacae strains isolated from patients and the environment C, clinical strain; E, environmental strain; PFGE, pulsed-field gel electrophoresis

The 11 *E. cloacae *and five *K. pneumoniae *strains from clinical and environmental sources (Table 3) were analyzed for clonality by PFGE using the XbaI enzyme. A dendrogram was generated using BioNumerics software. The five *K. pneumoniae *isolates exhibited four distinct PFGE profiles (A-D) (Figure 1). One representative strain from each profile was selected for WGS.

**Figure 2:**
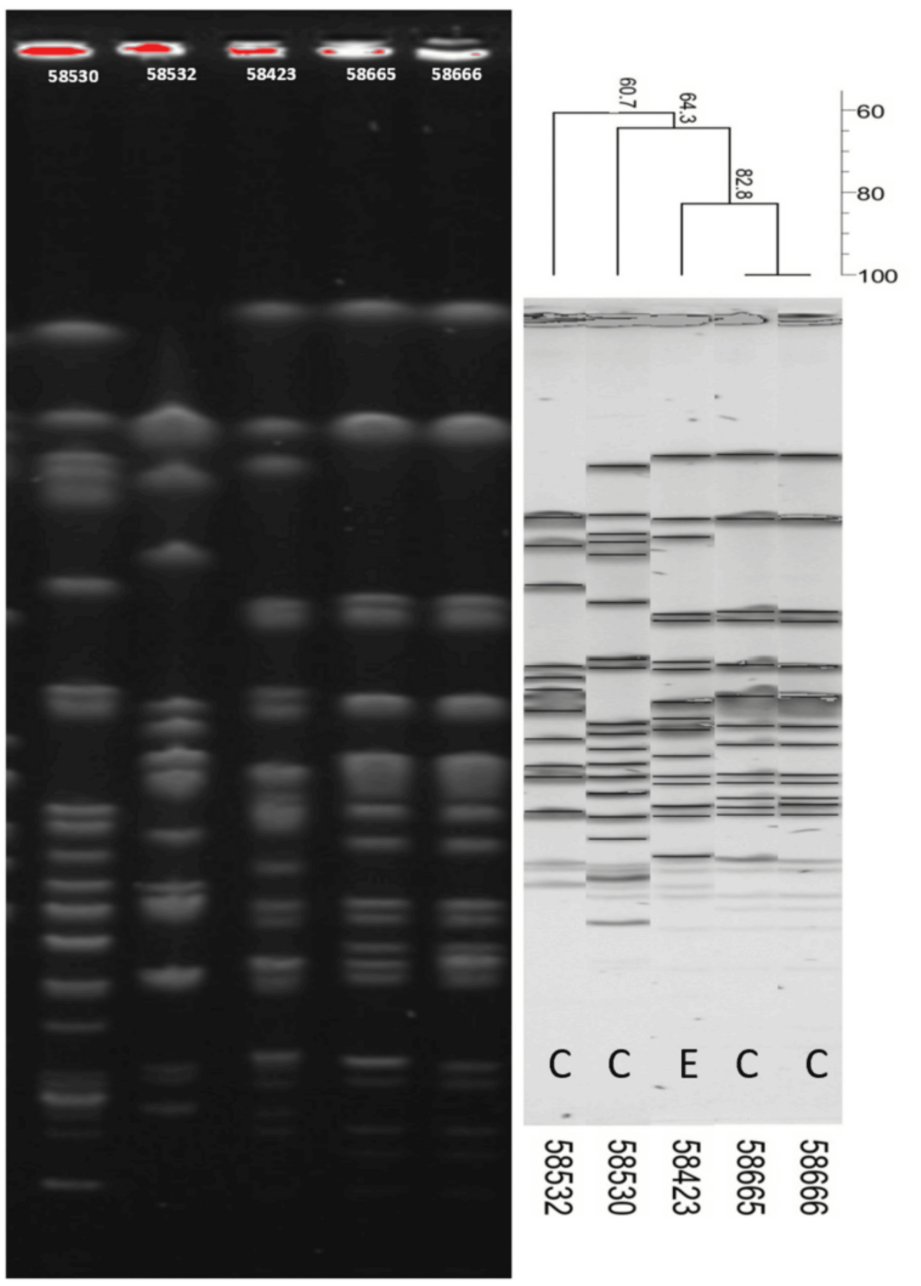
PFGE-based phylogeny of Klebsiella pneumoniae strains isolated from patients and the environment C, clinical strain; E, environmental strain; PFGE, pulsed-field gel electrophoresis

The diversity among *E. cloacae* strains was higher, with five distinct pulsotypes (E-I) identified (Figure 2). One environmental strain and two representative clinical strains were selected for WGS for further characterization.

Genetic diversity, resistance genes, and virulence factors in* K. pneumoniae*

**Figure 3:**
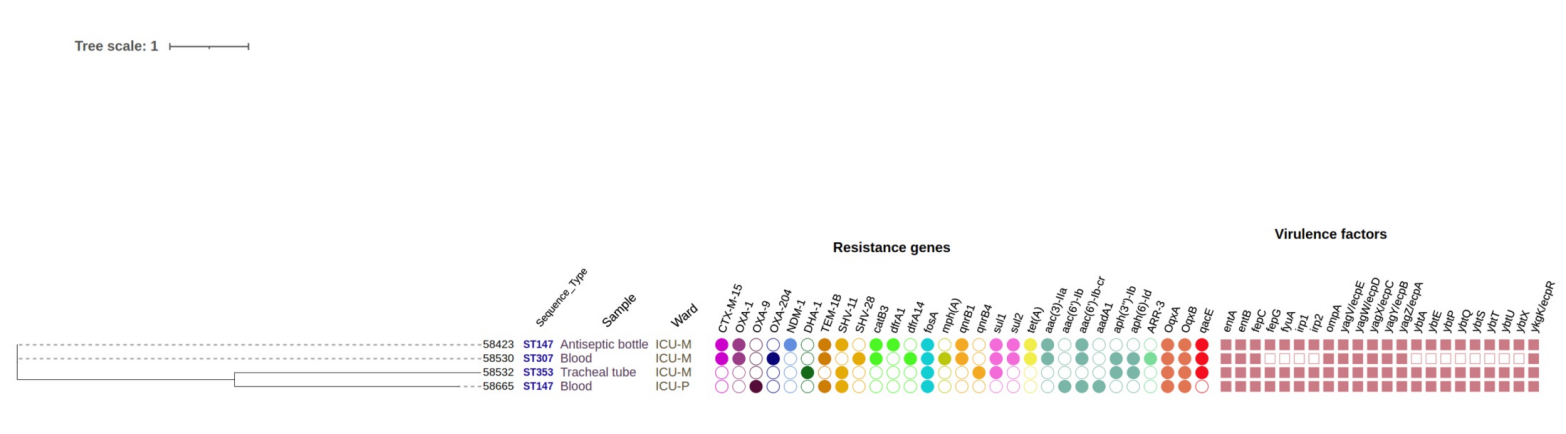
MLST-based phylogeny of Klebsiella pneumoniae isolates using iTOL MLST, multilocus sequence typing

Three STs were identified among the* K. pneumoniae* isolates. ST147 (n = 2) was shared between one clinical and one environmental strain; these isolates were not clonal, differing by 140 SNPs (Appendix A). Two additional STs were identified: ST307 and ST353 (Figure 3).

The environmental strain 58423 ST147 carried the carbapenemase NDM-1, the ESBL CTX-M-15, the non-ESBL SHV-11, and multiple resistance genes to non-β-lactam antibiotics. It also harbored the biocide resistance gene *qacE *(Figure 3). The clinical strain 58530 ST147 carried CTX-M-15, SHV-28, and the OXA-204 carbapenemase (OXA-48-like enzyme), while strain 58532 carried the extended-spectrum cephalosporinase gene *blaDHA*. Both strains also carried *qacE*. All *K. pneumoniae *strains possessed numerous virulence genes (Figure 3).

Genetic diversity and resistance genes in *E. cloacae*

Among the three fully sequenced *E. cloacae *isolates, two distinct STs were identified. The environmental isolate 58514, assigned to ST78, was recovered from a soap dispenser in ICU-P. This isolate carried the *blaACT-15 *gene and the *fosA *gene. The two other isolates belonged to ST344 and were recovered from clinical samples in two separate ICUs: one from a urinary catheter in ICU-M and the other from a blood sample in ICU-S. These two strains differed by only six SNPs, indicating that they were clonal (Appendix B). The ST344 isolates harbored the AmpC β-lactamase gene *blaACT-16 *and the ESBL gene *blaCTX-M-15*. Other β-lactamase genes were also detected, including *blaOXA-1*, along with multiple additional resistance determinants targeting aminoglycosides (*aph(6)-Id, aph(3'')-Ib, aac(3)-IIa, aac(6')-Ib-cr, aadA1*), fluoroquinolones (*qnrB1*), sulfonamides (*sul2*), tetracyclines (*tet(A)*), fosfomycin (*fosA*), phenicols (*catB3, catA1*), and trimethoprim (*dfrA14*).

## Discussion

HAIs represent a major global public health challenge, leading to serious complications, from sepsis to mortality, particularly in vulnerable patients such as those hospitalized in ICUs and newborns. According to WHO, the prevalence of HAIs is strongly influenced by a country's socioeconomic status. In high-income countries, approximately 7% of hospitalized patients develop an HAI, compared to more than 15% in low- and middle-income countries [5]. In Africa, HAI prevalence ranges from 7.24% to 28% [6], with Tunisia reporting one of the highest rates at 25.2% [7].

HAIs are frequently associated with MDR pathogens, particularly ESBL- and carbapenemase-producing Enterobacterales, which the WHO classifies as critical-priority pathogens due to their growing role in HAIs [8]. These bacteria are often waterborne and can be transmitted through fecally contaminated water or food. It was previously believed that this group survives poorly on environmental surfaces compared to other nosocomial pathogens. However, recent evidence suggests that *E. coli *and *Klebsiella *spp. can persist for over a year under desiccated conditions, while *Serratia marcescens *can remain viable for several months [9]. In multiple nosocomial outbreaks, environmental contamination has been linked to the transmission of pathogens. Environmental reservoirs, including surfaces, equipment, and inanimate objects, have been implicated in numerous nosocomial outbreaks. Inadequate hand hygiene among HCWs plays a key role in the transmission cycle, either through direct patient contact or indirect contact with contaminated surfaces. It is estimated that 20-40% of HAIs can be attributed to such cross-transmission via HCW hands [10].

Sahloul University Hospital, and particularly its ICUs, experiences recurrent outbreaks of MDR bacteria, notably involving *K. pneumoniae*, *Acinetobacter baumannii*, and *Proteus mirabilis *[11-14]. In 2020 alone, the microbiology diagnostic laboratory at Sahloul Hospital identified 450 isolates of *K. pneumoniae *and 175 of *E. cloacae*. Among the total 625 isolates, 62 (9.92%) originated from three ICUs: ICU-M, ICU-S, and ICU-P.

Of these 62 ICU-derived strains, 50% (31/62) exhibited resistance to third-generation cephalosporins (cefotaxime), and 29% (18/62) were resistant to carbapenems (ertapenem), highlighting a significant presence of multidrug resistance among ICU patients. In response to this concerning prevalence, a four-month surveillance study was conducted in 2020 to evaluate the potential role of the ICU environment, including surfaces, medical equipment, and HCWs, in the dissemination of resistant Enterobacterales (*K. pneumoniae *and *E. cloacae*).

A total of 140 environmental samples were collected across the three ICUs, of which only nine (6.42%) tested positive for resistant Enterobacterales, yielding 15 resistant isolates in total. Notably, eight of these were *E. cloacae*, with two isolates recovered from distinct soap dispensers in ICU-P and six others detected in ICU-S from various sources: HCWs (n = 2), a respirator (n = 1), a syringe plunger (n = 1), a patient bed (n = 1), and an electric switch (n = 1). A single *K. pneumoniae* isolate was identified in ICU-M, originating from an antiseptic solution bottle. These results confirm the potential role of HCWs (n = 2) in the transmission cycle of MDR Enterobacterales, with two *E. cloacae *strains directly isolated from the hands of staff in ICU-S, highlighting the importance of strict hand hygiene and infection control practices.

Although the rate of environmental contamination was relatively low, several critical contact surfaces and medical equipment items were contaminated with MDR Enterobacterales. This investigation represents the second study conducted at Sahloul Hospital focusing on the environmental dissemination of MDR pathogens. The first study, carried out in 2020-2021, documented the colonization of environmental surfaces, medical equipment, and HCWs’ hands by carbapenem-resistant *A. baumannii *[15], providing compelling evidence that both environmental reservoirs and inadequate hand hygiene practices were key contributors to patient contamination in ICUs.

Multilocus sequence typing analysis of four* K. pneumoniae* isolates fully sequenced and recovered between September and December 2020 from both clinical (n = 3) and environmental sources (n = 1) revealed three distinct STs. Two isolates belonged to ST147: one from a blood sample in ICU-P and the other from an antiseptic solution bottle in ICU-M. Despite sharing the same ST, these isolates were not clonal, differing by 140 SNPs. The two remaining isolates were identified as ST307 and ST353, both recovered from clinical samples (blood and tracheal aspirate, respectively) in ICU-M. No evidence of genetic relatedness was observed between clinical and environmental isolates collected from the same ward, suggesting the absence of direct transmission between the two sources during the study period.

Molecular characterization of the *K. pneumoniae *ST147 isolate recovered from the antiseptic solution bottle in ICU-M revealed the co-production of CTX-M-15 and NDM-1 enzymes, along with several additional resistance genes, including the biocide resistance gene *qacE*. ST147 is recognized as a high-risk clone implicated in numerous hospital outbreaks worldwide [16]. In Tunisia, considered an endemic region, this clone has frequently been associated with nosocomial outbreaks and is a major driver of antimicrobial resistance dissemination in healthcare settings, especially ICUs [17].

ST147 is believed to have emerged in the early 1990s, with global spread accelerating in the late 1990s and early 2000s. This dissemination was largely driven by mutations in the quinolone resistance-determining regions and the acquisition of the *blaCTX-M-15 *gene, often carried on IncF or IncR plasmids [17]. In the mid- to late 2000s, the global expansion of ST147 continued, facilitated by the acquisition of diverse carbapenemase genes across different regions [18]. In Tunisia, *K. pneumoniae *ST147 isolates have been predominantly associated with the spread of OXA-204 and OXA-48 carbapenemases [19]. However, the recent emergence of ST147 strains harboring *blaNDM-1 *marks a worrisome shift in their resistance landscape, indicating an ongoing evolutionary trend that amplifies their clinical impact and epidemiological risk [20,21]. The detection of a high-risk clone on an antiseptic solution bottle in an ICU is particularly alarming, highlighting the ability of this clone to persist and contaminate even supposedly sterile environments.

Another *K. pneumoniae *clone identified during this study was ST307, recovered from a blood sample in ICU-M. This isolate co-produced the ESBL CTX-M-15 and the carbapenemase OXA-204, highlighting its MDR profile. The ST307 clone was first identified in the Netherlands in 2008 and has since emerged as a high-risk, globally disseminated lineage. Globally, ST307 has been linked to the spread of several carbapenemase genes, including *blaNDM-1*, and is increasingly implicated in nosocomial outbreaks due to its adaptability and ability to accumulate resistance mechanisms [22]. In Tunisia, ST307 has been previously reported in isolates producing ESBLs and, more recently, in association with NDM-1. The detection of this clone in a bloodstream infection reinforces its clinical significance and underlines the ongoing evolution and diversification of resistance within this lineage. Its presence in the ICU setting raises concern about potential silent dissemination and the challenges it poses for infection control and antimicrobial stewardship efforts.

The biocide resistance gene *qacE *was identified in both clinical and environmental *K. pneumoniae *isolates. This co-resistance to biocides and antibiotics poses a growing challenge, as biocides are widely used in hospitals to prevent and control HAIs. While moderate biocide use can help reduce *K. pneumoniae *infections [23], the emergence of strains with co-resistance to antibiotics and biocides may limit the effectiveness of biocides in controlling hospital outbreaks and MDR bacterial transmission [24].

Among the three fully sequenced *E. cloacae *isolates, two distinct STs were identified. Two of the isolates belonged to ST344 and were recovered from clinical samples in two separate ICUs: one from a urinary catheter in ICU-M and the other from a blood sample in ICU-S. These two strains differed by only six SNPs, indicating that they were clonal. This high degree of genetic similarity strongly suggests possible inter-ICU transmission, either through patient transfer, shared medical equipment, or HCWs. These ST344 isolates harbored the AmpC β-lactamase gene *blaACT-16 *and the ESBL gene *blaCTX-M-15*, along with multiple additional resistance determinants targeting aminoglycosides, fluoroquinolones, sulfonamides, tetracyclines, and fosfomycin. The detection of such an MDR clone in distinct ICU wards highlights its potential for silent spread in the hospital environment.

Notably, *E. cloacae *has been increasingly recognized in Tunisia as an emerging nosocomial pathogen, particularly in high-risk hospital settings such as ICUs. The environmental *E. cloacae *isolate assigned to ST78 was recovered from a soap dispenser in ICU-P. This isolate carried the *blaACT-15* and *fosA *genes. ST78 is considered one of the high-risk clones within the *E. cloacae *complex (ECC), as it has been frequently reported worldwide in association with nosocomial infections. This lineage has been implicated in the dissemination of clinically important resistance genes, particularly among ESBL-producing ECC and carbapenem-resistant *E. cloacae*, highlighting its epidemiological significance [25-28]. The presence of this clone on a hygiene-related object such as a soap dispenser raises concerns about inadequate disinfection practices and the potential role of contaminated surfaces in the persistence and transmission of MDR *E. cloacae*.

The colonization of a soap dispenser by a high-risk *E. cloacae *clone ST78 and of an antiseptic bottle by a carbapenemase-producing *K. pneumoniae *ST147 strain, both commonly used items, illustrates the vulnerability of high-touch surfaces as potential reservoirs of MDR organisms. According to Huslage et al., “high-touch surfaces” are defined based on the frequency of contact with HCWs in the patient’s immediate environment and typically include objects such as bed rails, supply carts, overbed tables, and intravenous pumps [29]. Although often overlooked, items like soap dispensers or antiseptic solution bottles fall into this category due to repeated handling by HCWs. Such contaminated high-touch surfaces pose one of the greatest risks for HAI transmission [30].

Limitations

This study has several limitations that should be acknowledged. First, the survey was conducted over a limited period of four months, which may not fully capture temporal variations in the prevalence of MDR organisms and patterns of environmental contamination. Second, only a small subset of clinical and environmental strains was fully sequenced, which may have led to an underestimation of genetic diversity and transmission routes within the hospital. Third, environmental sampling was restricted to certain ICUs and surfaces and may not represent the full extent of contamination throughout the hospital or in other high-risk units. Finally, although the study highlights potential transmission pathways between the environment, HCWs, and patients, the lack of direct observational data on hand hygiene compliance or equipment disinfection practices limits our ability to confirm causal relationships. Future studies with larger sample sizes, longer surveillance periods, and real-time infection control assessments are warranted to better understand the transmission dynamics of MDR Enterobacterales in Sahloul Hospital.

## Conclusions

This study highlights the critical role of the hospital environment, particularly high-contact surfaces, in the persistence and transmission of high-risk clones of MDR Enterobacterales in ICUs at Sahloul Hospital. The detection of carbapenemase-producing *K. pneumoniae *ST147 on an antiseptic solution bottle and resistant *E. cloacae *ST78 on a soap dispenser underscores deficiencies in infection prevention practices and emphasizes the role of inadequate hand hygiene among HCWs in the spread of MDR pathogens. Although WGS revealed a 140-SNP difference between the environmental and clinical ST147 isolates, indicating that direct transmission did not occur during the study period, our findings suggest that environmental surfaces in ICUs can serve as reservoirs of high-risk Enterobacterales lineages, such as *K. pneumoniae *ST147 and *E. cloacae *ST78. The detection of clonally related *E. cloacae *strains in patients from different ICUs further suggests the potential for cross-transmission, highlighting the importance of ongoing molecular surveillance.

Furthermore, the co-occurrence of antibiotic and biocide resistance genes in these isolates raises concerns about the effectiveness of standard infection control measures and underscores the selective pressure imposed by widespread biocide use in healthcare settings. These findings call for enhanced antimicrobial resistance surveillance, the implementation of rigorous cleaning protocols targeting high-contact surfaces, and regular environmental monitoring to reduce the risk of MDR pathogen dissemination in hospital environments.

## Appendices

Appendix A 

**Table 4:** SNP distance matrix on Klebsiella pneumoniae MLST, multilocus sequence typing; SNP, single-nucleotide polymorphism

snp-dists 0.8.2	MLST	Identifier	58423	58530	58532	58665
	ST147	58423	0	22161	22846	140
	ST307	58530	22161	0	23186	22193
	ST353	58532	22846	23186	0	22882
	ST147	58665	140	22193	22882	0

Appendix B

**Table 5:** SNP distance matrix on Enterobacter cloacae MLST, multilocus sequence typing; SNP, single-nucleotide polymorphism

snp-dists 0.8.2	MLST	Identifier	58514	58531	58552
	ST78	58514	0	134808	134827
	ST344	58531	134808	0	6
	ST344	58552	134827	6	0
